# Exoscope-based supracerebellar infratentorial approach for a pineal meningioma in the prone position

**DOI:** 10.3171/2023.10.FOCVID23155

**Published:** 2024-01-01

**Authors:** Arka N. Mallela, Tritan J. Plute, Hussam Abou-Al-Shaar, David T. Fernandes Cabral, Constantinos G. Hadjipanayis

**Affiliations:** 1 Department of Neurological Surgery, University of Pittsburgh School of Medicine, Pittsburgh, Pennsylvania; and; 2 Hillman Cancer Center, Pittsburgh, Pennsylvania

**Keywords:** exoscope, pineal gland, quadrigeminal cistern, prone, intraoperative visualization, supracerebellar infratentorial

## Abstract

The supracerebellar infratentorial (SCIT) approach is a well-described corridor to lesions in the quadrigeminal cistern, pineal gland, and dorsal midbrain. It can be performed in the prone or sitting position. The sitting position offers the benefit of gravity retraction of the cerebellum but comes at the expense of nonergonomic hand positioning and the potential risk of air embolism. The 3D exoscope is an alternative to the operating microscope and permits the SCIT approach in the prone position with excellent visualization. This video demonstrates exoscope-based SCIT approach for resection of a pineal meningioma in the prone position.

The video can be found here: https://stream.cadmore.media/r10.3171/2023.10.FOCVID23155

## Transcript

Exoscope-based supracerebellar infratentorial approach for a pineal tumor in the prone position.

### 0:27 Background and Objective.

The supracerebellar infratentorial approach is a well-described surgical corridor to lesions in the quadrigeminal cistern, pineal gland, and dorsal midbrain.^1–3^ It can be performed in the prone or sitting position. The 3D exoscope is an alternative to the operating microscope that may improve visualization and surgeon comfort.^4–6^ Here we demonstrate our experience with the exoscope in SCIT for resection of a pineal region meningioma performed in the prone position.

### 0:55 Case Presentation.

The patient is a 27-year-old female with prior history of craniospinal irradiation for childhood acute lymphoblastic leukemia, presenting with progressive headaches, found to have hydrocephalus and a pineal region mass on imaging. She is neurologically intact except for headaches and developmental delay.

### 1:12 Preoperative Imaging.

On T1-weighted MRI with contrast, we see a homogeneously enhancing lesion in the pineal region that is displacing the internal cerebral veins superiorly and the basal veins of Rosenthal laterally.

### 1:24 Positioning and Technique.

The patient is positioned prone with a military chin head tuck. The exoscope is positioned above the midline. The screen is placed in front of the patient’s head directly in the operator’s field of view. The positioning of the exoscope as well as the screen allow for the operator to work in a comfortable position with neutral hand positioning. The supracerebellar infratentorial approach is otherwise performed in a standard fashion.

### 1:50 Craniotomy.

A suboccipital craniotomy has been performed.

### 1:55 Dural Opening and Supracerebellar Dissection.

The dura has been opened in a C-shaped fashion and the supracerebellar dissection proceeds.

The thick arachnoid at the tentorial apex is opened.

### 1:48 Tumor Visualized.

After coagulation and cutting of the precentral cerebellar vein, the tumor is visualized. Frozen section was sent and then the tumor was internally debulked. Due to the firm nature of the tumor, ultrasonic aspiration was used to perform the internal debulking.

### 2:43 Lateral Dissection.

Once sufficient internal debulking had been achieved, the lateral aspects of the tumor were teased off the structures in the quadrigeminal cistern with particular care with regard to the many veins in this region.

The superior attachments to the internal cerebral veins were identified and sharply divided.

Once all attachments to the lateral and superior structures had been removed, the tumor was carefully teased out of the quadrigeminal cistern and removed from the patient.

### 4:12 Tumor Inspected.

We inspected the tumor once it had been removed from the patient and confirmed that it had been removed in its entirety.

### 4:24 Vein of Galen and Quadrigeminal Cistern.

After meticulous hemostasis had been achieved, we inspected the quadrigeminal cistern. The exoscope provides a beautiful view of the contents of the quadrigeminal cistern including the vein of Galen as well as the posterior aspect of the third ventricle.

### 4:38 Closure.

The closure was performed in the usual fashion.

### 4:42 Postoperative Imaging.

Postoperative imaging revealed gross-total resection of the tumor.

### 4:51 Postoperative Outcome.

Postoperatively, the patient had a ventriculostomy placed, which was subsequently weaned and removed. Following this, her headaches had resolved. The patient was then discharged to inpatient rehab. Final pathology demonstrated WHO grade I meningioma with Ki-67 of less than 1%.

### 5:06 Conclusions.

In summary, the exoscope provides an excellent visualization of critical structures in the quadrigeminal cistern while performing the supracerebellar infratentorial approach in the prone position. The high range of motion for exoscope permits very steep trajectories in both this approach and other approaches. This obviates the need for gravity retraction from the sitting position, which comes at the expense of nonergonomic hand positioning. Further research will continue to expand the use of the exoscope in the neurosurgical operating room.

## Disclosures

Dr. Hadjipanayis reported personal fees from Synaptive Medical during the conduct of the study, and personal fees from Stryker Corp., Hemerion Therapeutics, and Integra outside the submitted work.

## Author Contributions

Primary surgeon: Hadjipanayis. Assistant surgeon: Mallela. Editing and drafting the video and abstract: all authors. Critically revising the work: all authors. Reviewed submitted version of the work: all authors. Approved the final version of the work on behalf of all authors: Mallela. Supervision: Hadjipanayis.

## Supplemental Information

### Patient Informed Consent

The necessary patient informed consent was obtained in this study.
